# Functional FGFR4 Gly388Arg polymorphism contributes to oral squamous cell carcinoma susceptibility

**DOI:** 10.18632/oncotarget.21958

**Published:** 2017-10-23

**Authors:** Chia-Hsuan Chou, Ming-Ju Hsieh, Chun-Yi Chuang, Jen-Tsun Lin, Chia-Ming Yeh, Pao-Yu Tseng, Shun-Fa Yang, Mu-Kuan Chen, Chiao-Wen Lin

**Affiliations:** ^1^ Institute of Medicine, Chung Shan Medical University, Taichung, Taiwan; ^2^ Department of Medical Research, Chung Shan Medical University Hospital, Taichung, Taiwan; ^3^ Cancer Research Center, Changhua Christian Hospital, Changhua, Taiwan; ^4^ Graduate Institute of Biomedical Sciences, China Medical University, Taichung, Taiwan; ^5^ School of Medicine, Chung Shan Medical University, Taichung, Taiwan; ^6^ Department of Otolaryngology, Chung Shan Medical University Hospital, Taichung, Taiwan; ^7^ Division of Hematology and Oncology, Department of Medicine, Changhua Christian Hospital, Changhua, Taiwan; ^8^ Department of Otorhinolaryngology-Head and Neck Surgery, Changhua Christian Hospital, Changhua, Taiwan; ^9^ Institute of Oral Sciences, Chung Shan Medical University, Taichung, Taiwan; ^10^ Department of Dentistry, Chung Shan Medical University Hospital, Taichung, Taiwan

**Keywords:** FGFR4, polymorphism, OSCC, metastasis, betel quid chewing

## Abstract

Aberrations of the fibroblast growth factor receptor 4 (FGFR4) genomic region include amplification of *FGFR4*, activation of *FGFR4* kinase domain mutations, and overexpression of FGFR4, which lead to sustained cell proliferation and contribute to tumor development. However, the association between *FGFR4* single-nucleotide polymorphisms (SNPs) and risk of oral squamous cell carcinoma (OSCC) remains to be determined. We investigated the relationships between *FGFR4* genetic polymorphisms, OSCC development and clinicopathological variables. We recruited a total of 955 patients with OSCC and 1191 controls. Four SNPs of *FGFR4* (rs2011077, rs351855, rs7708357, and rs1966265) were examined using real-time polymerase chain reaction. We found that with the rs351855 GA genotype and a combination of the GA and AA genotypes exhibited a 1.431-fold (95% CI: 1.092-1.876) and 1.335-fold (95% CI: 1.033-1.725) higher risk of OSCC. However, patients with OSCC with a homozygous A/A genotype of *FGFR4* rs351855 polymorphism had a lower risk of advanced stage OSCC (P = 0.0252). Furthermore, the patients with the *FGFR4* rs351855/rs1966265 A-A haplotype had a 2.890-fold (95% confidence interval [CI]: 2.257–3.700) higher risk of OSCC than the controls. Betel quid chewers with the A-A haplotype had a considerably higher risk (95% CI: 16.159–26.937) of OSCC than did betel quid nonchewers with other haplotypes. Moreover, an additional integrated in silico analysis proposed that rs351855 G allele variant to the A allele exhibited a relatively low energy of the transmembrane region. In conclusion, our results suggest that the *FGFR4* rs351855 may play a role in susceptibility for OSCC development.

## INTRODUCTION

Fibroblast growth factor receptors (FGFRs) modulate some crucial biological processes, such as cell proliferation, cell differentiation and tissue repair [1–3]. In humans, four FGFR family members (FGFR1 to FGFR4), which act as transmembrane tyrosine kinase receptors [4], and 18 fibroblast growth factors (FGFs), which are ligands for FGFRs, are observed. In cancers, aberrations of the FGFR4 genomic region include the amplification of *FGFR4*, activation of *FGFR4* kinase domain mutations, and overexpression of FGFR4, which lead to sustained cell proliferation and contribute to tumor development [3].

Genetic variants of FGFR4 with several diseases have been documented. Gao et al observed that the A allele of rs351855 in FGFR4 was associated with a higher risk and worse prognosis of non-Hodgkin's lymphoma than were other alleles [5]. Additional studies have revealed that four SNPs of *FGFR4*, namely rs2011077 (T/C), rs351855 (G/A, Gly388 to Arg388), rs7708357 (G/A), and rs1966265 (A/G, Ile10Val), might affect protein expression [6–8]. Our previous study revealed that *FGFR4* rs351855 might be related with the risk of hepatocellular carcinoma (HCC) associated with liver cirrhosis and might increase the alpha-fetoprotein level in Taiwanese patients with HCC [9]. Another of our previous studies revealed that *FGFR4* rs2011077 and rs1966265 were associated with the progression of normal cervical tissues to precancerous lesions in Taiwanese women, and FGFR4 rs351855 was associated with poor patient survival [10].

**Figure 1:**
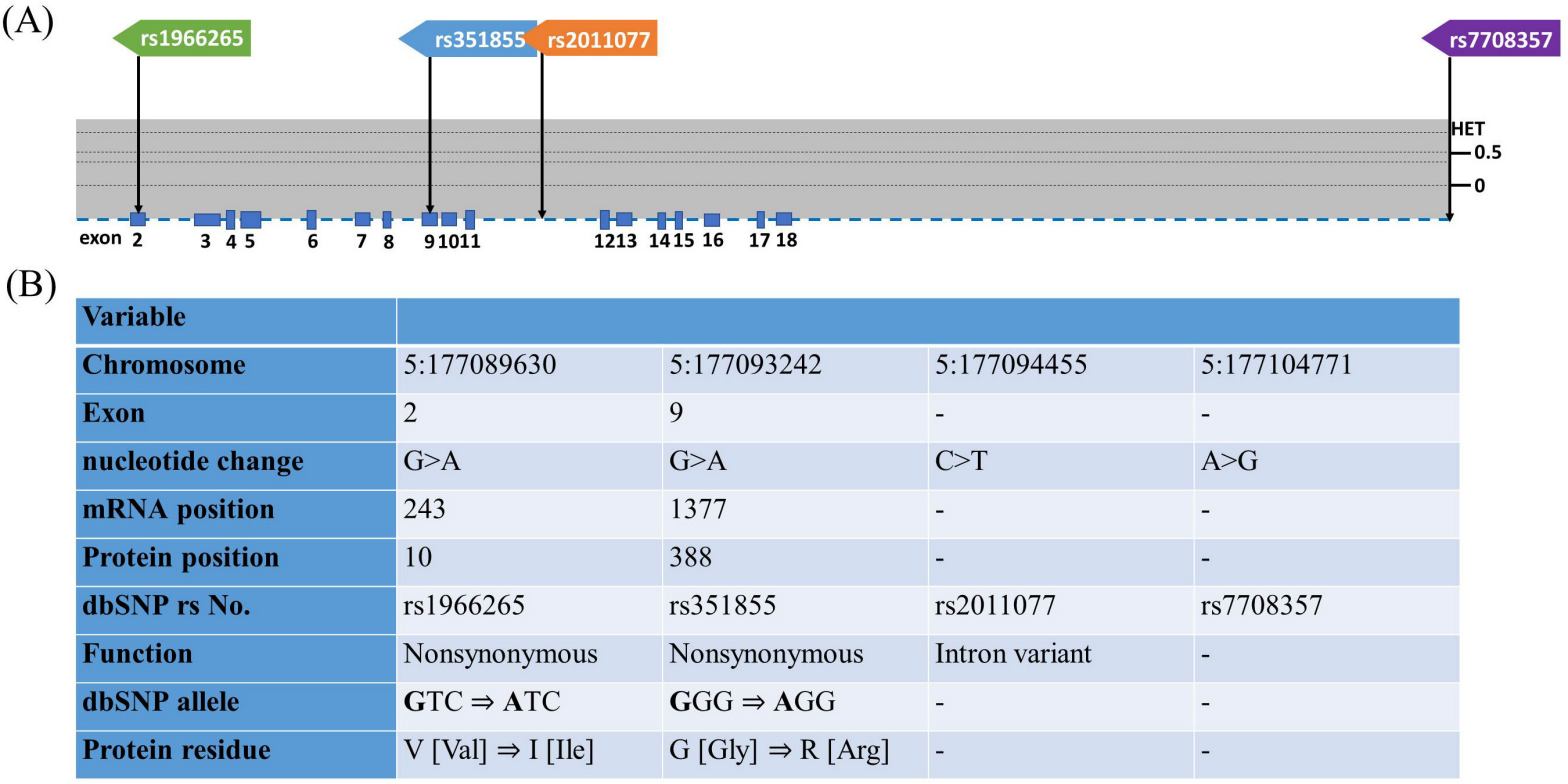
Exon and intron position of *FGFR4* gene in human and *FGFR4* gene polymorphisms assessed in study **(A)** The position of four SNPs of *FGFR4* gene from the chromosome chr 5:177089630 to 177104771 (reference genome GRCh38.p7). The lower panel shows population-specific heterozygosity frequencies of this polymorphism in East Asian population (HAPMAP-CHB). **(B)** FGFR4gene polymorphisms assessed in this study.

OSCC represents the most common oral neoplasm, and more than of 90% of all oral neoplasms are estimated to be OSCCs [11]. OSCC has the fourth highest incidence of malignancy in males of Taiwan [12]. Most cases of OSCC are diagnosed late [13]. At least 50% of patients with OSCC presented with late stage tumors during their first visit to medical centers in Taiwan, which resulted in a low overall 5-year survival rate [14]. In Taiwan, cigarette smoking, betel quid chewing, and alcohol consumption are the major risk factors for OSCC [15]. Betel quid chewing is a crucial risk factor because nearly 2.5 million people chew betel quid in Taiwan. Consequently, the incidence rate of OSCC in Taiwan is relatively high. However, only a few studies have investigated the associations between polymorphisms of *FGFR4*, environmental carcinogens and OSCC susceptibility, and the distribution of clinical features of OSCC in the Taiwanese male population. Therefore, we designed a case-control study to identify four *FGFR4* gene polymorphisms (rs1966265, rs351855, rs2011077, and rs7708357; Figure 1A and 1B) and analyze their contribution to OSCCs as well as to determine the relationships between environmental factors and the clinicopathological characteristics of OSCC.

## RESULTS

### Population statistics and data of the participants

The statistical analysis of the demographic characteristics of the participants is shown in Table 1. In total, our data recruited 2146 participants in this case–control study, comprising 955 male patients with OSCC and 1191 controls. This study found significantly different distributions of betel quid chewing (p < 0.001), cigarette smoking (p < 0.001), and alcohol drinking (p < 0.001) between the patients with OSCC and controls.

**Table 1:** The distributions of demographical characteristics in 1191 controls and 955 male patients with oral cancer

Variable	Controls(N=1191)	Patients(N=955)	p Value
Age (yrs)	Mean ± S.D.	Mean ± S.D.	
	53.92 ± 10.03	54.76 ± 11.01	p=0.068
Betel quid chewing			
No	992 (83.3%)	188 (19.7%)	
Yes	199 (16.7%)	767 (80.3%)	p <0.001*
Cigarette smoking			
No	558 (46.9%)	109 (11.4%)	
Yes	633 (53.1%)	846 (88.6%)	p <0.001*
Alcohol drinking			
No	954 (80.1%)	424 (44.4%)	
Yes	237 (19.9%)	531 (55.6%)	p <0.001*
Stage			
I+II		467 (48.9%)	
III+IV		488 (51.1%)	
Tumor T status			
T1+T2		551 (57.7%)	
T3+T4		404 (42.3%)	
Lymph node status			
N0		648 (67.9%)	
N1+N2+N3		307 (32.1%)	
Metastasis			
M0		944 (98.9%)	
M1		11 (1.1%)	
Cell differentiation			
Well differentiated		151 (15.8%)	
Moderately or poorly differentiated		804 (84.2%)	

Mann-Whitney U test or Fisher's exact test was used between healthy controls and patients with oral cancer. * *p* value < 0.05 as statistically significant.

### FGFR4 gene polymorphism in patients with OSCC and controls

The genotypic and allelic frequencies of *FGFR4* in patients with OSCC and controls are listed in Table 2. In the control group, the frequencies of the alleles of *FGFR4* exhibited Hardy–Weinberg equilibrium (p>0.05). After adjustment for several variables, the data shown that participants with the rs351855 GA genotype and a combination of the GA and AA genotypes exhibited a 1.431-fold (95% CI: 1.092–1.876) and 1.335-fold (95% CI: 1.033–1.725) higher risk of OSCC, respectively, than wild-type homozygous participants.

**Table 2:** Genotyping and allele frequency of *FGFR4* single nucleotide polymorphism (SNP) in oral cancer and normal controls

Variable	ControlsN=1191 (%)	PatientsN=955 (%)	AOR (95% CI)	p Value
**rs2011077**				
TT	326 (27.4%)	236 (24.7%)	1.000 (reference)	
TC	577 (48.5%)	509 (53.3%)	1.188 (0.907-1.557)	p=0.210
CC	288 (24.1%)	210 (22.0%)	0.986 (0.716-1.359)	p=0.933
TC+CC	865 (72.6%)	719 (75.3%)	1.121 (0.868-1.448)	p=0.380
T allele	1229 (51.6%)	981 (51.4%)	1.000 (reference)	
C allele	1153 (48.4%)	929 (48.6%)	0.998 (0.852-1.170)	p=0.985
**rs351855**				
GG	334 (28.0%)	225 (23.5%)	1.000 (reference)	
GA	596 (50.0%)	524 (54.9%)	1.431 (1.092-1.876)	p=0.009*
AA	261 (22.0%)	206 (21.6%)	1.136 (0.821-1.572)	p=0.443
GA+AA	857 (72.0%)	730 (76.5%)	1.335 (1.033-1.725)	p=0.027*
G allele	1264 (53.1%)	974 (51.0%)	1.000 (reference)	
A allele	1118 (46.9%)	936 (49.0%)	1.076 (0.918-1.260)	p=0.367
**rs7708357**				
GG	1167 (98.0%)	932 (97.6%)	1.000 (reference)	
GA	23 (1.9%)	22 (2.3%)	1.327 (0.624-2.820)	p=0.462
AA	1 (0.1%)	1 (0.1%)	0.647 (0.022-18.961)	p=0.800
AG+AA	24 (2.0%)	23 (2.4%)	1.283 (0.614-2.683)	p=0.507
G allele	2357 (98.9%)	1886 (98.7%)	1.000 (reference)	
A allele	25 (1.1%)	24 (1.3%)	1.241 (0.606-2.542)	p=0.555
**rs1966265**				
AA	326 (27.4%)	228 (23.9%)	1.000 (reference)	
AG	580 (48.7%)	514 (53.8%)	1.227 (0.936-1.610)	p=0.139
GG	285 (23.9%)	213 (22.3%)	1.020 (0.739-1.408)	p=0.905
AG+GG	865 (72.6%)	727 (76.1%)	1.159 (0.896-1.499)	p=0.260
A allele	1232 (51.7%)	970 (50.8%)	1.000 (reference)	
G allele	1150 (48.3%)	940 (49.2%)	1.015 (0.866-1.189)	p=0.857

The adjusted odds ratios (AORs) with their 95% confidence intervals (CIs) were estimated by multiple logistic regression models after controlling for betel quid chewing, cigarette smoking and alcohol drinking.

### Combined effects of environmental factors and FGFR4 gene polymorphism on OSCC

Our study determined the combined effect of environmental factors and *FGFR4* gene SNPs on OSCC susceptibility (Table 3). In the study population, among 1479 smokers who were also betel quid chewers, participants with at least one C allele of rs2011077, one A allele of rs351855, one A allele of rs7708357, or one G allele of rs1966265 exhibited 4.267-fold (95% CI: 2.855–6.376), 7.624-fold (95% CI: 4.839–12.011), 4.004-fold (95% CI: 1.931–8.300), and 4.354-fold (95% CI: 2.905–6.526) higher risks of OSCC, respectively, than smokers with the wild-type genes who were betel quid nonchewers.

**Table 3:** Associations of the combined effect of *FGFR4* gene polymorphisms and betel quid chewing with the susceptibility to oral cancer among 1479 smokers

Variable	Controls(n=633) (%)	Patients(n=846) (%)	OR (95% CI)	p Value	AOR (95% CI)	p Value
**rs2011077**						
^a^TT genotype & non-betel quid chewing	111 (17.5%)	39 (4.6%)	1.00 (reference)		1.000 (reference)	
^b^TC or CC genotype or betel quid chewing	171 (27.0%)	189 (22.3%)	**3.146 (2.068-4.785)**	p<0.001*	**2.684 (1.736-4.149)**	p<0.001*
^c^TC or CC genotype with betel quid chewing	351 (55.5%)	618 (73.1%)	**5.011 (3.401-7.384)**	p<0.001*	**4.267 (2.855-6.376)**	p<0.001*
**rs351855**						
^a^GG genotype & non-betel quid chewing	127 (20.1%)	26 (3.1%)	1.00 (reference)		1.000 (reference)	
^b^GA or AA genotype or betel quid chewing	147 (23.2%)	199 (23.5%)	**6.612 (4.121-10.067)**	p<0.001*	**5.581 (3.427-9.091)**	p<0.001*
^c^GA or AA genotype with betel quid chewing	359 (56.7%)	621 (73.4%)	**8.449 (5.433-13.137)**	p<0.001*	**7.624 (4.839-12.011)**	p<0.001*
**rs7708357**						
^a^GG genotype & non-betel quid chewing	437 (69.0%)	118 (14.0%)	1.00 (reference)		1.000 (reference)	
^b^GA or AA genotype or betel quid chewing	181 (28.6%)	710 (83.9%)	**14.527 (11.193-18.854)**	p<0.001*	**12.137 (9.303-15.834)**	p<0.001*
^c^GA or AA genotype with betel quid chewing	15 (2.4%)	18 (2.1%)	**4.444 (2.175-9.082)**	p<0.001*	**4.004 (1.931-8.300)**	p<0.001*
**rs1966265**						
^a^AA genotype & non-betel quid chewing	111 (17.5%)	38 (4.5%)	1.00 (reference)		1.000 (reference)	
^b^AG or GG genotype or betel quid chewing	168 (26.5%)	183 (21.6%)	**3.182 (2.083-4.861)**	p<0.001*	**2.726 (1.756-4.232)**	p<0.001*
^c^AG or GG genotype with betel quid chewing	354 (56.0%)	625 (73.9%)	**5.157 (3.489-7.623)**	p<0.001*	**4.354 (2.905-6.526)**	p<0.001*

The adjusted odds ratios (AORs) with their 95% confidence intervals (CIs) were estimated by multiple logistic regression models after controlling for alcohol drinking.

p values were adjusted for multiple comparisons by applying the Bonferroni correction.

### Effects of the polymorphic genotypes of FGFR4 on the clinical status of OSCC

Furthermore, our study explored the effects of the polymorphic genotypes of *FGFR4* on the clinicopathological status of OSCC, which includes TNM clinical staging, tumor size, lymph node involvement, and cell differentiation (Table 4). No significant associations were observed between the rs2011077, rs7708357, and rs1966265 gene polymorphisms and the clinicopathological status of OSCC. However, among the 955 patients with oral cancer, those who had a polymorphic rs351855 (A/A) gene received protection against developing an advanced clinical stage (stage III/IV) of OSCC (OR: 0.648; 95% CI: 0.443–0.947) compared with patients with the rs351855 wild type after adjustment for several variables (adjusted OR: 0.637; 95% CI: 0.435–0.933).

**Table 4:** Genotyping frequency of *FGFR4* rs351855 polymorphism on clinical statuses with oral cancer

Clinical stage			OR (95% CI)	p Value	AOR (95% CI)^a^	p Value
*FGFR4* rs351855	Stage I/II (n=467) n (%)	Stage III/IV (n=488) n (%)				
GG	98 (21.0%)	127 (26.0%)	1.000 (reference)		1.000 (reference)	
GA	257 (55.0%)	267 (54.7%)	0.802 (0.585-1.098)	p=0.168	0.797 (0.581-1.093)	p=0.159
AA	112 (24.0%)	94 (19.3%)	0.648 (0.443-0.947)	p=0.025*	0.637 (0.435-0.933)	p=0.021*
**Tumor size**						
*FGFR4* rs351855	<T2 (n=551) n (%)	> T2 (n=404) n (%)				
GG	122 (22.1%)	103 (25.5%)	1.000 (reference)		1.000 (reference)	
GA	304 (55.2%)	220 (54.5%)	0.857 (0.626-1.174)	p=0.337	0.860 (0.628-1.178)	p=0.348
AA	125 (22.7%)	81 (20.0%)	0.768 (0.523-1.126)	p=0.176	0.767 (0.522-1.126)	p=0.175
**Lymph node metastasis**						
*FGFR4* rs351855	No (n=648) n (%)	Yes (n=307) n (%)				
GG	148 (22.8%)	77 (25.1%)	1.000 (reference)		1.000 (reference)	
GA	353 (54.5%)	171 (55.7%)	0.931 (0.669-1.296)	p=0.672	0.928 (0.666-1.292)	p=0.658
AA	147 (22.7%)	59 (19.2%)	0.771 (0.513-1.161)	p=0.213	0.765 (0.508-1.153)	p=0.201
**Metastasis**						
*FGFR4* rs351855	M0 (n=944) n (%)	M1 (n=11) n (%)				
GG	221 (23.4%)	4 (36.4%)	1.000 (reference)		1.000 (reference)	
GA	520 (55.1%)	4 (36.4%)	0.425 (0.105-1.715)	p=0.229	0.415 (0.102-1.680)	p=0.218
AA	203 (21.5%)	3 (27.2%)	0.816 (0.181-3.692)	p=0.792	0.783 (0.172-3.559)	p=0.751
**Cell differentiation**						
*FGFR4* rs351855	Well (n=151) n (%)	Moderate/poor (n=804) n (%)				
GG	33 (21.9%)	192 (23.9%)	1.000 (reference)		1.000 (reference)	
GA	87 (57.6%)	437 (54.4%)	0.863 (0.559-1.334)	p=0.580	0.865 (0.559-1.338)	p=0.515
AA	31 (20.5%)	175 (21.7%)	0.970 (0.570-1.651)	p=0.911	0.982 (0.577-1.674)	p=0.948

^a^ Adjusting for the effects of betel quid chewing, cigarette smoking and alcohol drinking.

### Haplotype analysis of polymorphisms in FGFR4 in the susceptibility to OSCC

Our study used PHASE version 2.1 to reconstruct the common haplotypes. The haplotype frequencies of rs351855/rs1966265 among the study participants are presented in Table 5. When participants with the rs351855 G allele and rs1966265 A allele were selected as a reference group, participants with the G-G (AOR = 2.750; 95% CI: 2.150–3.516) and the A-A (adjusted OR = 2.890; 95% CI: 2.257–3.700) haplotypes were significantly associated with the risk of OSCC.

**Table 5:** Frequencies of *FGFR4* haplotypes in OSCC patients and control subjects

Haplotype block		Controls	Patients	OR (95% CI)	p Value	AOR (95% CI)^a^	p Value
rs351855 G/A	rs1966265 A/G	n = 2382	n = 1910				
G	A	527 (22.1%)	204 (10.7%)	1.000 (reference)		1.000 (reference)	
G	G	737 (30.9%)	770 (40.3%)	2.699 (2.231-3.266)	p<0.001*	2.750 (2.150-3.516)^b^	p<0.001*
A	G	413 (17.3%)	170 (8.9%)	1.063 (0.836-1.353)	p=0.672	1.002 (0.739-1.360)	p=0.987
A	A	705 (29.7%)	766 (40.1%)	2.807 (2.318-3.398)^b^	p<0.001*	2.890 (2.257-3.700)^b^	p<0.001*

^a^ Adjusting for the effects of betel quid chewing, cigarette smoking and alcohol drinking.

### Combined effects of betel quid chewing and FGFR4 haplotypes on OSCC development

Our study further analyzed the relationship between the combined effect of betel quid chewing and the *FGFR4* haplotypes on OSCC development (Table 6). Betel quid nonchewers with other haplotypes (G-A, G-G, and A-G) were considered the reference group. After adjustment for several variables, we observed that participants who were betel quid nonchewers with the A-A haplotype had a 1.960-fold (95% CI: 1.560–2.464) higher risk of OSCC than the reference group. Betel quid chewers with other haplotypes had a 15.394-fold (95% CI: 12.446–19.039) higher risk of OSCC than did the reference group. Overall, betel nut chewers with the A-A haplotype had the highest risk of OSCC development (AOR: 20.863; 95% CI: 16.159–26.937).

**Table 6:** Combined effect of betel quid chewing and *FGFR4* haplotypes on OSCC development

Betel quid chewing	*FGFR4* haplotype	Controls	Patients	AOR (95% CI)^b^	p Value
		n = 2382	n = 1910		
Yes	A-A	124 (5.2%)	596 (31.2%)	20.863 (16.159-26.937)	p<0.001*
Yes	Others^a^	274 (11.5%)	938 (49.1%)	15.394 (12.446-19.039)	p<0.001*
No	A-A	581 (24.4%)	170 (8.9%)	1.960 (1.560-2.464)	p<0.001*
No	Others^a^	1403 (58.9%)	206 (10.8%)	1.000 (reference)	

^a^ Other haplotypes included G-A, G-G, and A-G.

^b^ Adjusting for the effects of cigarette smoking and alcohol drinking.

P-values were adjusted for multiple comparisons by applying the Bonferroni correction.

### Functional connotation of the FGFR4 rs351855 locus

**Figure 2:**
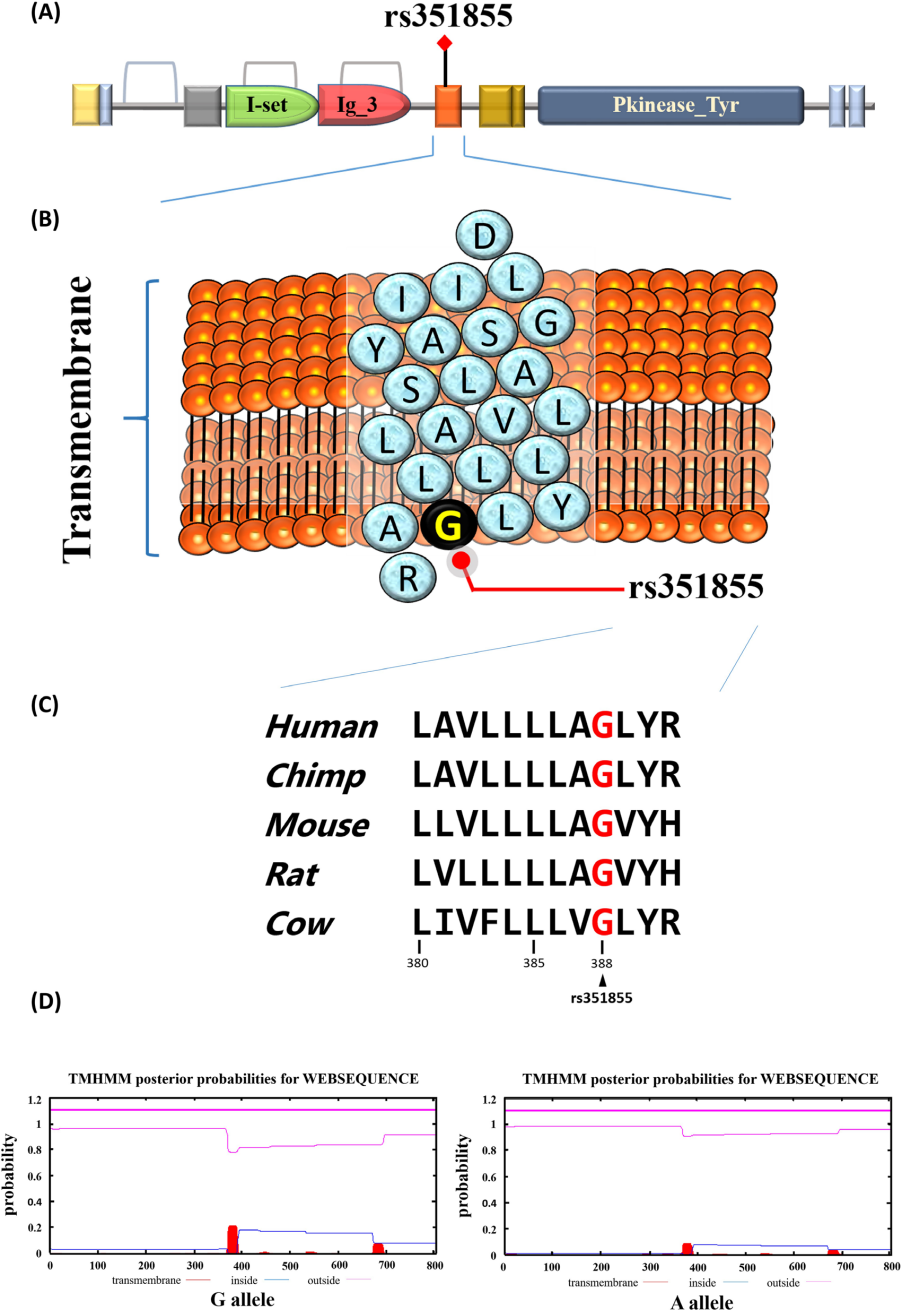
Functional implication and *In silico* profiling of *FGFR4* SNP rs351855 **(A)** Schematic representation of the full-length human FGFR4 protein domain organization. The orange represent the transmembrane that rs351855 was located in this region. **(B)** Ribbon diagram depicts the transmembrane of rs351855. The blue circles represent amino acids abbreviation and the black circle represents the rs351855 residue change. **(C)** Mammalian of FGFR4 proteins sequences showed in this alignment. Human (*homo*, *NM_002011.4*), chimp (*Pan troglodytes, NC_006472.4*), mouse (*mus*, *NM_008011.2*), rat (*rattus norvegicus*, *NM_001109904.1*), and cow (*bos taurus, NM_001109904.1*). **(D)** Prediction of transmembrane helices in rs351855.

Our study also conducted a functional analysis of *FGFR4* rs351855. The data obtained the *FGFR4* rs351855 locus in the transmembrane domain from the Pfam database (Figure 2A and 2B). The Gly388Arg variation at the rs351855 locus is a highly conserved sequence across the human, chimp, mouse, and rat genomes (Figure 2C). Then, TMHMM Server v. 2.0 was used to predict of transmembrane helices in proteins. As shown in Figure 2D, the rs351855 G allele variant to the A allele exhibited a relatively low energy of the transmembrane region.

### Association of expression of FGFR4 and clinicopathological characteristics in OSCC

**Figure 3:**
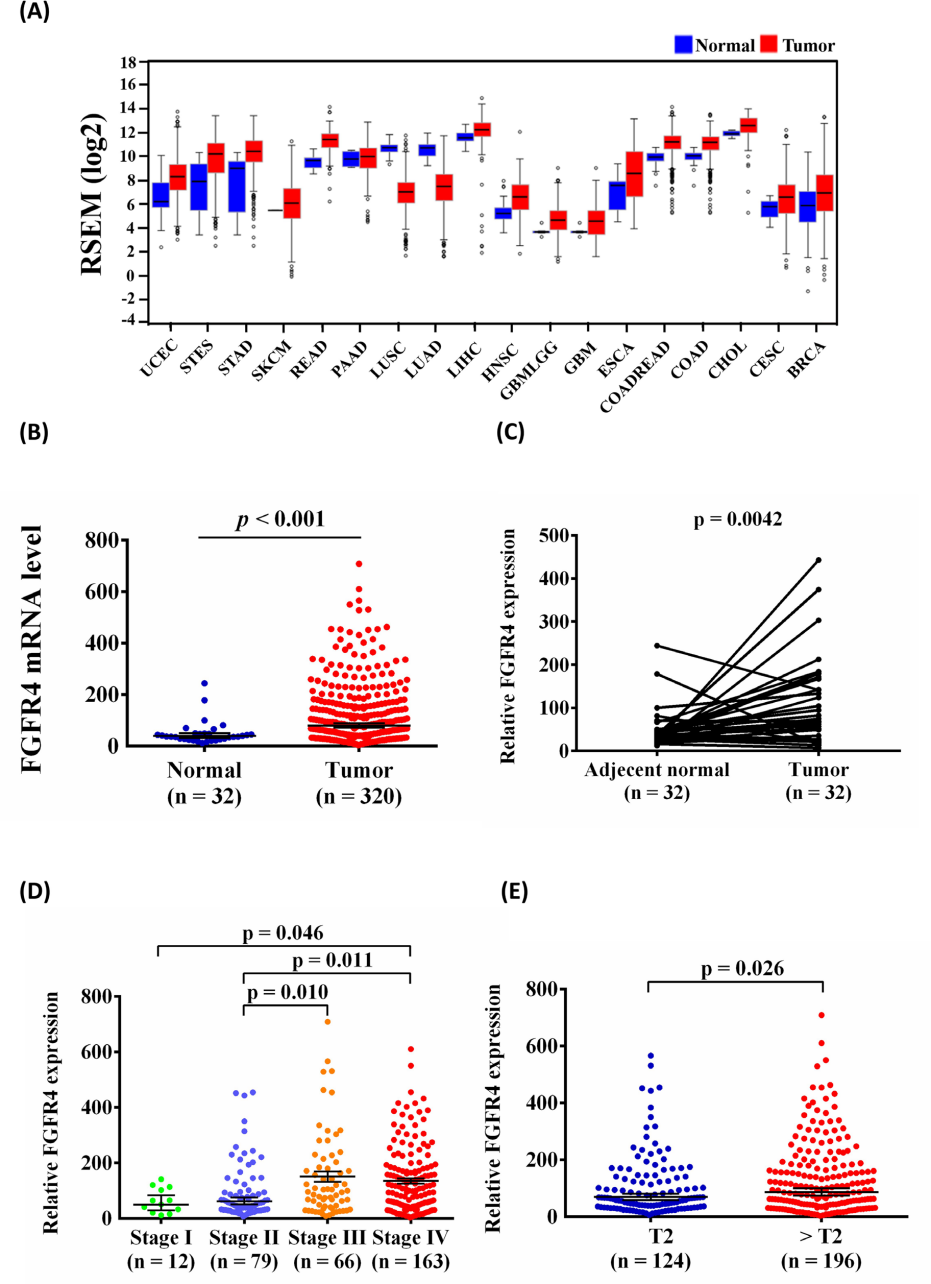
FGFR4 mRNA levels increased in OSCC samples **(A)** Different cancer types of FGFR4 mRNA level from The Cancer Genome Atlas (TCGA) Data Portal from Broad GDAC Firehose data portal. **(B)** The expression of FGFR4 in normal and OSCC from TCGA Data Portal. **(C)** Relative expression of FGFR4 in 32 pairs of OSCC tumor tissues and their corresponding adjacent non-cancerous tissues. **(D)** Relative FGFR4 levels were compared according to clinical stage. **(E)** Relative FGFR4 levels were compared according to tumor T status.

To further support our findings, we evaluated FGFR4 expression by using The Cancer Genome Atlas (TCGA) Data Portal from Broad GDAC Firehose to determine whether FGFR4 was involved in the development of head and neck squamous cell carcinoma (HNSCC). We found that FGFR4 mRNA expression was higher in tumor tissues than in normal tissues in various cancers (Figure 3A). Furthermore, we chose patients with OSCC from the HNSCC database and found that FGFR4 expression significantly increased in cancerous tissues compared with normal tissues (Figure 3B). Moreover, FGFR4 mRNA expression was also significantly higher in the OSCC tissue than in their corresponding noncancerous tissue (Figure 3C). Otherwise, advanced clinical stage (stage III/IV) of OSCC patients had significantly higher expression of FGFR4 than stage I/II (Figure 3D). The relative FGFR4 mRNA level was significantly higher in > T2 than in ≦ T2 (Figure 3E).

## DISCUSSION

In this study, we found that patients with *FGFR4* SNP rs351855 with heterozygous GA and a combination of rs351855 GA and AA had an increased risk of OSCC. Moreover, the combined effect of environmental factors and *FGFR4* polymorphisms significantly increased the risk of OSCC. Notably, patients with *FGFR4* SNP rs351855 with homozygous AA were less likely to develop stage III or IV cancers. We further observed that the combination of betel quid chewing and *FGFR4* haplotype A-A had the highest risk of OSCC.

Dysregulation of FGFR4 activity has been observed in human epithelial carcinomas including head and neck, thyroid, breast, hepatocellular, and prostate tumors [16, 17]. A study showed that during treatment with doxorubicin or cyclophosphamide, the aberrant expression of FGFR4 in cancer cells causes reduced apoptosis sensitivity [18]. The previous study showed that overexpression of FGFR4 is significantly associated with a high clinical stage and tumor grade as well as poor patient survival in prostate cancer [19]. Overexpression of FGFR4 has been associated with resistance to chemotherapy in patients with breast cancer [18]. However, little information is available on the role of FGFR4 in OSCC. In the present study, we observed that *FGFR4* rs351855 G/A (Gly388Arg) polymorphisms were significantly associated with susceptibility to OSCC (Table 2). Heinzle et al [20] demonstrated that among patients with colorectal cancer, *FGFR4* A allele carriers had a five-fold higher risk of tumors that were stage II or higher. Chen et al [21] reported that patients with the *FGFR4* SNP rs351855 AA or AG genotype exhibited a poorer biochemical recurrence-free survival than did those with the GG genotype. Therefore, the SNP rs351855 of *FGFR4* polymorphism might provide a basis for surveillance programs. Moreover, the interaction between the *FGFR4* polymorphisms investigated and environmental factor was significant (betel nut chewing and tobacco use) and was associated with a high incidence of OSCC development. These results suggest that *FGFR4* polymorphisms exhibit synergistic effects with betel nut chewing and tobacco smoking, on susceptibility to OSCC.

The present study revealed the protective role of *FGFR4* rs351855 AA genotype against developing an advanced clinical stage (stage III/IV); OR: 0.648; 95% CI: 0.443–0.947) cancer (Table 4). However, Sheu et al [9] observed that patients with HCC carrying at least one A genotype (GA and AA) of the *FGFR4* rs351855 polymorphism may have an increased risk of liver cirrhosis. In patients with OSCC, Choi et al [22] demonstrated that with the *FGFR4* allele Arg/Arg or Arg/Gly at amino acid 388 were associated with advanced N stage (pathologic N2+N3) when compared with a Gly/Gly allele-carrying group. In patients with HNSCC, Streit et al [23] revealed that high expression levels of FGFR4 and the Arg388 allele were associated with poor clinical outcomes. However, Ansell et al [24] reported contradictory results; they found that patients carrying the *FGFR4* rs351855 Gly allele had a significantly higher risk of HNSCC. However, the opposite results from different studies on the same cancer merit further investigation.

Betel quid chewing has been established as a critical determinant of OSCC [12, 25]. The habit of chewing betel has resulted in a high incidence rate of OSCC in Taiwan [12]. Arecoline, which is a major component of areca nut, can produce 3-methyl nitrosamine propionitrile, a potent carcinogen, and safrole-like DNA adducts that have been shown to be genotoxic and mutagenic [26]. Several studies have reported that the ingredients of betel quid are correlated with carcinogenic effects and tumor promotion [27–31]. In the present study, we assessed the combined effects of betel quid chewing and *FGFR4* haplotypes among patients with OSCC. Participants who were betel nut chewers and had a high-risk haplotype were at a higher risk of OSCC than were those who were either betel nut chewers or had the high-risk haplotype. OSCC is etiologically related to betel nut chewing, which can trigger and aggravate the risk of OSCC. When the rs351855 A allele of *FGFR4* polymorphism was substituted with the G allele, lower transmembrane domain energy was observed (Figure 2D). Bange et al [32] and Morimoto et al [33] have demonstrated that the Arg^388^ allele of *FGFR4* polymorphism in the transmembrane domain is associated with poor prognosis in breast cancer, colon cancer and high-grade soft-tissue sarcoma, respectively. Besides, a study found that the FGFR4-R388 allele was linked to poor cancer prognosis and this risk variant enhanced pericellular ECM degradation by membrane type 1 matrix metalloproteinase (MT1-MMP) in a polarized manner, which resulted in rapid tumor cell invasion in collagen [34]. The T-A haplotype is a key factor in the course of the OSCC progression and in the synergistic effects with environmental factors, such as betel nut chewing, that intensify the risk of OSCC.

We further investigated expression of FGFR4 in patients with OSCC recruited from patients with HNSCC in the TCGA database. We observed that the expression of FGFR4 was significantly higher in cancerous tissues than that in the normal tissue in OSCC (Figure 3B). Koole et al [35] revealed that the FGFR4 protein was overexpressed in OSCC cells. Shi et al [36] established that FGFR4 was high expressed in nasopharyngeal carcinoma (NPC) clinical samples and cell lines. The results concluded that high expression of FGFR4 was associated with poor prognosis of NPC patients. We also observed that FGFR4 expression was higher in advanced clinical stages (stage III/IV) than stage I/II (Figure 3D). A previous study found that high expression of the *FGFR4* Arg388 allele was significantly associated with reduced overall survival and with an advanced tumor stage in HNSCC; data supporting our findings is available in the TCGA database [23]. In addition, various studies also showed the association between *FGFR4* Gly388Arg polymorphism and survival in HNSCC [22–24, 37–41]. Dutra et al. [41] found that Arg388 allele was correlated with lymphatic embolization and disease related premature death in squamous cell carcinoma of the mouth and oropharynx. da Costa Andrade et al. [37] confirmed that the *FGFR4* Arg388 allele had a significantly shorter survival in HNSCC. Farnebo et al. [38] illustrated that the *FGFR4* Arg388 allele had a significantly longer overall survival in HNSCC. These conflicting evidences on *FGFR4* Gly388Arg polymorphism in HNSCC need to be further investigated and clarified the mechanism.

Our study had some limitations. First, our data were obtained from only two medical centers; thus, referral bias might have occurred. Second, the questionnaire information on betel nut chewing, cigarette smoking, and alcohol consumption was reported as ‘‘frequent’’ or ‘‘never.’’ Therefore, we could not comprehensively analyze the amount betel nut, tobacco, and alcohol used, length of use, and history of betel nut chewing, cigarette smoking, and alcohol consumption. Another limitation of our study is functional assay and the mechanism should be elucidated in laboratory and clinically.

In conclusion, the rs351855 polymorphism of *FGFR4* has potential predictive significance in OSCC. A combination of betel nut chewing and the *FGFR4* T-A haplotype is associated with an increased risk of OSCC. High expression levels of the FGFR4 were significantly associated with a clinical advanced stage. The rs351855 (Gly388Arg) might be used to improve the prediction of clinical prognosis of patients with OSCC.

## MATERIALS AND METHODS

### Study population

We recruited 955 male patients with OSCC from Chung Shan Medical University Hospital in Taichung, Taiwan, between 2007 and 2016. We selected 1191 healthy male individuals without a history of cancer at any sites to constitute the control group from Taiwan Biobank. Personal information and characteristics were obtained from the participants. Furthermore, demographic characteristics; details of betel quid chewing, tobacco smoking, and alcohol consumption; and medical histories of the participants were recorded. Patients’ medical information, including tumor–nodes–metastasis (TNM) clinical staging, primary tumor size, lymph node involvement, and histologic grade, was obtained from their medical records. The patients were staged clinically at the time of diagnosis according to the TNM staging system of the American Joint Committee on Cancer [42]. The study was approved by the Institutional Review Board of Chung Shan Medical University Hospital (CSMUH No: CS13214-1). Whole-blood specimens from the patients with OSCC and controls were collected in tubes containing ethylene diamine tetra-acetic acid (EDTA), immediately centrifuged, and stored at −80°C.

### Determination of genotypes

Genomic DNA was extracted from peripheral blood leukocytes using QIAamp DNA blood mini kits (Qiagen, Valencia, CA, USA) by following the manufacturer's instructions. We dissolved the extracted DNA in a Tris–EDTA buffer (10 mM Tris, 1 mM EDTA; pH 7.8) and subsequently quantified it by measuring at the absorbance at 260 nm. The final preparation was stored at −20°C and was then used to create templates for polymerase chain reaction (PCR). Allelic discrimination for the *FGFR4* SNPs was assessed using the TaqMan assay (ID C_8817791_10 for rs2011077, C_3166614_10 for rs351855, C_11270571_10 for rs7708357, and C_11317464_20 for rs1966265) with an ABI StepOne™ Real-Time PCR System (Applied Biosystems, Foster City, CA, USA) Subsequent assessment was performed using SDS version 3.0. The total volume of the TaqMan assay mixture was 10 μL, consisting of 5 μL of Master Mix, 0.25 μL of probes, and 10 ng of genomic DNA. The real-time PCR reaction included an initial denaturation step at 95°C for 10 min, followed by 40 amplification cycles of 95°C for 15 sec and 60°C for 1 min.

### Bioinformatics analysis

We used several bioinformatic tools to assess the putative functional relevance of the rs351855 *FGFR4* polymorphism. Data from the Pfam 31.0 database were used to identify the rs351855 polymorphism in sequence alignments (template as PDB accession number: 4TYE). We used UCSC Cancer Genomics Browser [43] for analyzing the molecular features of *FGFR4*. Furthermore, we used the TMHMM transmembrane domain of rs351855 with Server v. 2.0 to predict transmembrane helices in FGFR4.

### Statistical analysis

Genotype distributions of the four tagging single nucleotide polymorphisms (tSNPs) were tested for Hardy-Weinberg equilibrium (HWE), which means the allelic distribution between all populations and in our study was not different (*p* < 0.05). The Mann-Whitney U test and Fisher's exact test were used to compare differences in the distribution of age and demographic characteristics between the controls and OSCC patients. The adjusted odds ratios (ORs) and 95% confidence intervals (CIs) of the association between genotypes frequencies and risk plus clinicopathological characteristics were estimated by multiple testing (bonferroni correction), after controlling for other covariates. We estimated the common haplotypes by PHASE version 2.1 [44]. The data were analyzed by using SAS statistical software (Version 9.4; SAS Institute Inc., Cary, NC).
